# Supplementary material for: Understanding the needs of women undergoing breast ultrasound: Are male radiologists still needed?
Source: PLoS One. 2023 Nov 8;18(11):e0291007. doi: 10.1371/journal.pone.0291007 (PMC10631629; doi:10.1371/journal.pone.0291007)
Supplement: S1 Checklist — (DOCX) [file pone.0291007.s001.docx]

***PLOS ONE* Clinical Studies Checklist**

***PLOS ONE* manuscript number: ______**PONE-D-23-14827**_________________**

| **Complete the following if your study involved human participants or human subjects’ data. These questions should be addressed for prospective and retrospective studies.** | | |
| --- | --- | --- |
| 1. | Did you obtain ethics approval for this study?   - If yes, please upload (file type “Other”) the original approval document you received from your ethics committee. If the original document is in another language, please also provide an English translation.   ___ Uploaded __x_ N/A   - If you did not obtain ethical approval, please explain why this was not required.  \| **This is a qualitative Study. I am also a medical doctor and an psychologist. In Switzerland is this a situation, where is a ethical approval not necessary. I describe this rules in the manuscript.** \| \| --- \| |  |
| 2. | If your study involved human participants, please report in the Methods section when participants were recruited to the study.  __x_ Completed __ N/A |  |
| 3. | If you are reporting a study of medical records or archived samples, please report in the Methods section the date range in which human subjects’ data/samples were collected and the date(s) when you conducted this study.  __x_ Completed ___ N/A |  |
| 4. | Please specify in the Methods section whether authors had access to information that could identify individual participants during or after data collection.  _x__ Completed ___ N/A |  |
| 5. | If you are reporting an observational study – i.e. cohort, case-control, and cross-sectional studies – we recommend that the work is reported as per the requirements of the STROBE guidelines, and that you provide a completed STROBE checklist as a Supporting Information file with your submission.  The STROBE checklist was developed to improve the reporting of observational human subjects research, and is available here: <http://strobe-statement.org/fileadmin/Strobe/uploads/checklists/STROBE_checklist_v4_combined_PlosMedicine.docx>.  ___ Completed __x_ N/A |  |
| 6. | Please ensure that the author list and Corresponding Author entered in Editorial Manager match the author list and Corresponding Author in your manuscript file.  __x_ Completed |  |
