# Supplementary material for: Understanding the needs of women undergoing breast ultrasound: Are male radiologists still needed?
Source: PLoS One. 2023 Nov 8;18(11):e0291007. doi: 10.1371/journal.pone.0291007 (PMC10631629; doi:10.1371/journal.pone.0291007)
Supplement: Attachment — Submitted filename: Review notes.docx [file pone.0291007.s002.docx]

I am pleased to recommend accepting the submitted article for publication. The study presents the results of original research, providing valuable insights into the topic. The findings reported in this study have not been published elsewhere, which adds to the existing body of knowledge. **My only concern is the large number of authors, I would like to know what their contributions were.**

The experiments, statistics, and other analyses conducted in this research are of good technical standard. The authors have described these methods with sufficient detail, allowing for reproducibility and understanding of the study design. The data presented in the article support the conclusions drawn by the authors in an appropriate and logical manner.

One notable strength of this article is its clear and intelligible presentation. The authors have effectively communicated their research in standard English, making it accessible to a wide range of readers. The article also adheres to the ethical standards of experimentation and research integrity, demonstrating the authors' commitment to conducting their study in an ethically responsible manner.

In summary, this article fulfills the essential criteria for acceptance. It presents original research with good findings, good technical standards, appropriate conclusions supported by data, clear and accessible language, adherence to ethical standards, and compliance with reporting guidelines. Therefore, I recommend accepting this article for publication.
